# Closing the loop between horizon scanning and health technology assessment – an overview of topics submitted for appraisal in England

**DOI:** 10.1017/S0266462323000491

**Published:** 2023-09-11

**Authors:** Sarah Khalid Khan, Sonia G. Gonzalez-Moral, Kate Lanyi, Dapo Ogunbayo, Dawn Craig

**Affiliations:** NIHR Innovation Observatory, Population Health Sciences Institute, Newcastle University, Newcastle upon Tyne, UK

**Keywords:** biomedical technology assessment, health technology assessment, health service, health technology, horizon scanning

## Abstract

**Objectives:**

Horizon scanning for health technology appraisal (HTA) in England involves topic notification to the National Institute for Health and Care Excellence (NICE) via technology briefings. This activity is undertaken by the Innovation Observatory with submission timelines designed to ensure that HTA decisions align with regulatory approval time. In this paper, we aimed to track and assess the progression and current status of the topics notified for HTA and provide a descriptive analysis of these topics.

**Methods:**

Technology briefings were mapped from submission to NICE technology appraisal/highly specialized technologies recommendations from April 2017 until October 2021. This was done using a combination of searches on Google and NICE website, searching a downloadable spreadsheet containing NICE topic selection decisions, and querying NICE Topic Selection team. Analysis was undertaken regarding type of indications and interventions of submitted topics and published guidance.

**Results:**

Six-hundred and ninety-three topics entered the NICE scoping process, of which 94 percent were prioritized. As of November 2021, approximately 39 percent of prioritized topics were in scoping/in progress, 31 percent were proposed/completed, 20 percent were suspended/terminated, and 4 percent were referred back to Innovation Observatory (IO) for further monitoring.

**Conclusions:**

Our work demonstrates that horizon scanning for HTA is a complex and time-intensive process. Timelines and progress through HTA is challenging due to the growing number of innovative medicines, significant uncertainties, and limited transparency in clinical development and regulatory pathways. A better understanding of clinical trials and regulatory requirements may help eliminate some of this uncertainty and improve timely HTA.

## Introduction

Horizon scanning is an analytical method used in future forecasting to detect early signals of important development that takes a systematic approach to the examination of information sources (1). In the health and social care field, these signals may be described as new or emerging technologies. Horizon scanning usually follows a process of signal identification, filtration, prioritization, assessment, and dissemination (1). The outputs generated as part of this dissemination form the basis of the early awareness and alert (EAA) notification system. Horizon scanning is gaining recognition as an essential part of the health technology appraisal (HTA) process nationally and internationally (2). In the United Kingdom (UK), the National Institute for Health and Care Excellence (NICE) performs HTA for innovative medicines on behalf of the National Health Service (NHS) in England and Wales, and produces evidence-based guidance and advice on appropriate “topics” for health, public health and social care practitioners. In this context a “topic” is an innovative medicine and indication. Once guidance has been provided on relevant topics they become part of the range of medicines available to patients through the NHS.

**Figure 1.**
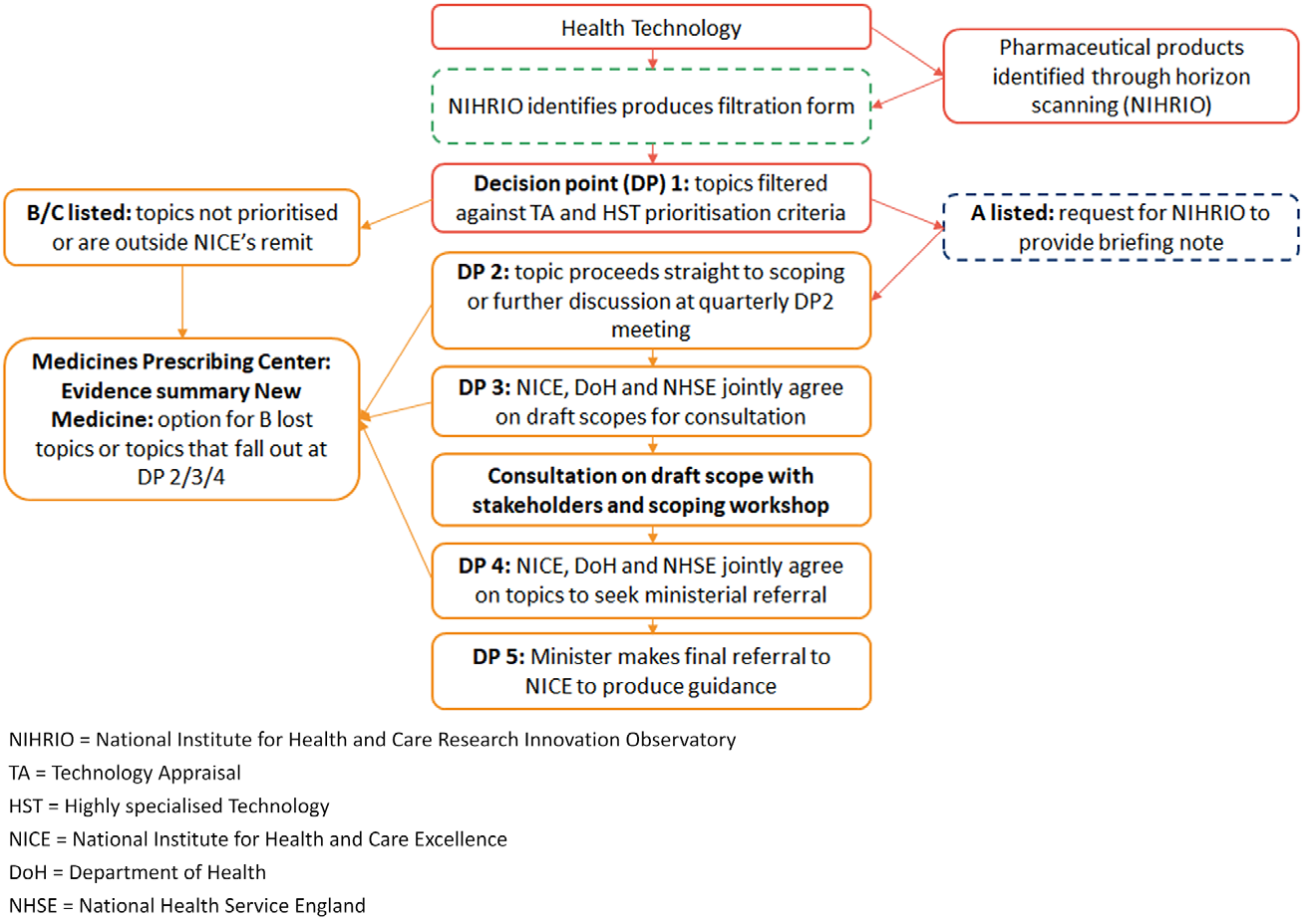
Overview of the NICE technology appraisal decision process and NIHR IO horizon-scanning inputs.

Horizon scanning and topic selection (TS) are integral steps in the HTA process of the NICE technology appraisal (TA)/highly specialized technologies (HST) program for innovative medicines. NICE TS involves timely identification, notification, prioritization, and scoping of potential topics that eventually results in formal referral of appropriate topics for HTA (Figure 1) (3). Horizon scanning in England is undertaken by the National Institute for Health and Care Research (NIHR) Innovation Observatory, the national horizon scanning, intelligence, and research center based at Newcastle University. The Innovation Observatory’s horizon scanning system identifies and notifies (filters) potential topics that meet the NICE TS remit (3). Figure 1 presents an overview of how the Innovation Observatory’s horizon scanning processes feed into the NICE HTA.

Until November 2021, topics identified by the Innovation Observatory were submitted to NICE TS via a two-stage signal process: Initial notification uses a *filtration form* to submit a topic that is anticipated to receive regulatory approval and/or launched in the EU/UK within a three-year timeframe. During Decision Point (DP) 1 a decision to progress the topic (A listed) may be recommended and a Technology Briefing (TB) is requested and produced prior to regulatory approval and/or launch in the EU/UK (3). Topics listed as B or C at DP1 are out of scope for NICE TA/HST. From DP2 to DP4 topics may be de-prioritized and routed out of the TA/HST. Until November 2021, TBs were issued at 17–20 months before anticipated regulatory approval and/or launch in the EU/UK (4). Since November 2021, the Innovation Observatory and NICE TS have agreed on a new timeframe for TB submission that extends the process up to 24 months ahead of anticipated regulatory approval and/or UK launch as a more realistic and achievable timeframe.

According to the published NICE process and methods guide for TA, the minimum time taken from the initiation of the TA process, once a formal referral is received, to the publication of the final TA document, which then becomes the TA guidance, is 245 calendar days for a Single-Technology Appraisal (STA). This process may be shorter for Fast Track Appraisal (FTA) or longer if an appraisal consultation document is deemed necessary by the appraisal committee (5).

One TA may contain one or more appraisal recommendations. These recommendations may fall into one of the following categories. A technology may be (i) recommended; (ii) optimized; (iii) only in research; (iv) not recommended or (v) recommended for use in the Cancer Drug Fund (CDF) (5).

When NICE recommends that a treatment be funded by the NHS, the regulations require that the period within which the health service must comply will be stated in the recommendations as 3 months, except when particular barriers to implementation within that period are identified (6).

Early alert and submission of potential topics on innovative medicines is essential for timely HTA and efficient patient access. The Innovation Observatory’s horizon scanning system aims to identify innovative and potentially disruptive topics (new or repurposed medicines) that are within 3- to 5-year timeframe of regulatory approval or launch in the UK/EU (7). For the purpose of this paper, we refer to new medicines as those that are not licensed for any indication in the UK and repurposed medicines are those that have an existing license and are now in clinical trials for a new indication, stage or line of treatment. However, the increasing numbers of innovative medicines, significant uncertainties, and limited transparency in clinical development and regulatory pathways make the forecasting of timelines and progression of topics through HTA challenging. One initiative the UK has put in place to ease some of the forecasting issues mentioned above and to act as the first step in securing market access for new medicines was the creation of the UK PharmaScan, a database of information on new medicines, indications, and formulations in the pharmaceutical pipeline entered by pharmaceutical companies. It is the primary source of information used by all of the UK’s national horizon scanning organizations, including the Innovation Observatory, NHS England and NHS Improvement, Scottish Medicines Consortium, and All Wales Medicines Strategy Group (8).

Topics identified from horizon scanning and submitted for potential HTA contain data that can provide valuable insights into emerging trends, gaps, and unmet needs in innovation. Furthermore, an analysis of these data can help the Innovation Observatory and other horizon scanning agencies achieve a more effective and efficient horizon scanning and EAA process. In this paper, we examine the topics that the Innovation Observatory has submitted to NICE via TBs for consideration within the HTA process. We specifically focus on topics that were filtered between April 2017 and October 2021, which represents the period between the Innovation Observatory’s inception and the change in the NICE TS notification timeline and submission process. This study aims to:

- Track and assess the progression and status of the topics notified to NICE TS for the TA/HST program to identify factors that can help refine and improve horizon scanning methods in topic identification for HTA;
- Provide a descriptive analysis of the topics that may help inform emerging trends and gaps in innovation areas, technology types, and clinical/therapy areas.

## Methods

### Horizon scanning database

The Innovation Observatory undertakes routine horizon scanning as part of its core function, utilizing a robust methodology to identify and track innovative (new or repurposed) medicines. Primary horizon scanning sources used include clinical trial registries, news media and press releases, and company pipeline meetings. Once an innovative medicine has been identified, UK PharmaScan is checked, and where a record exists, used to further validate and expand our intelligence. The Innovation Observatory maintains a comprehensive Medicines Innovation Database (MInD), focusing on medicines with potential UK/EU license/launch within ~5 years. The information collated from the horizon-scanning sources is extracted into MInD as individual “technology records” or “topics” via combination of semi-automated and manual processes by members of the horizon-scanning team. Each technology record holds information about the name and type of intervention/innovation of the medicines, the target therapy area/indication (including the line of treatment and population subgroups such as age, genetic mutations, or comorbidities), associated clinical trials and regulatory timelines, designations, and awards. The Innovation Observatory uses MInD to monitor potential topics that meet the NICE TS remit, by gathering intelligence on their clinical and regulatory timelines by active company engagement via regular pipeline meetings and email correspondence and through the use of UK PharmaScan, then producing TBs for those that are within a window of 17 to 20 month of anticipated UK/EU regulatory approval/launch.

### Search strategy and data collection

The Innovation Observatory’s MInD was queried to identify all topics with a TB submitted to the NICE TS team between April 2017 and October 2021. Each TB is assigned an Innovation Observatory internal identification (NIHRIO ID) number, in addition to the NICE TS ID number. Both IDs were used to match topics with the outcomes of a NICE TS decision using a spreadsheet that is publicly available on the NICE website (9). The NICE TS decisions state whether or not each topic will progress for a potential TA and the rationale behind this decision. Where some topics were missing and/or could not be identified on the spreadsheet using the relevant IDs, the following steps were undertaken:

- The spreadsheet was searched by intervention name(s) and target indication(s) and compared and matched where possible with the relevant topic(s)
- A fuzzy search for the topic(s) using the intervention name(s) was performed on both the NICE website and on the Google search engine to potentially identify scoping or related documents to help with the matching to relevant topics
- MInD was queried for some of the topics that could not be found using the above-mentioned steps to identify any topics that NICE had returned to the Innovation Observatory for monitoring.
- Following these steps, a small proportion of topics that could not be identified were queried with the NICE TS team to identify any relevant information about the status and/or progression, or lack of, of the topic

Key data points captured in the spreadsheet included relevant IDs, status on MInD, intervention name(s), indication/therapeutic area information, regulatory status, key innovation type, NICE TS decision, NICE Appraisal Stage (as of November 2021), estimated TA publication, if suspended or terminated (reasons provided).

### Approach to data synthesis/analysis

Descriptive data analysis was undertaken by three independent researchers and was checked for accuracy through consultation at regular meetings. Whilst undertaking these analyses, topics referred to NICE did not remain static in the NICE TA processes, so the team of researchers updated the statuses of the referred topics regularly to maximize accuracy. The analyses presented in this study are those last undertaken in November 2021.

## Results

### Overview of progression and status of topics submitted to NICE TS

**Figure 2.**
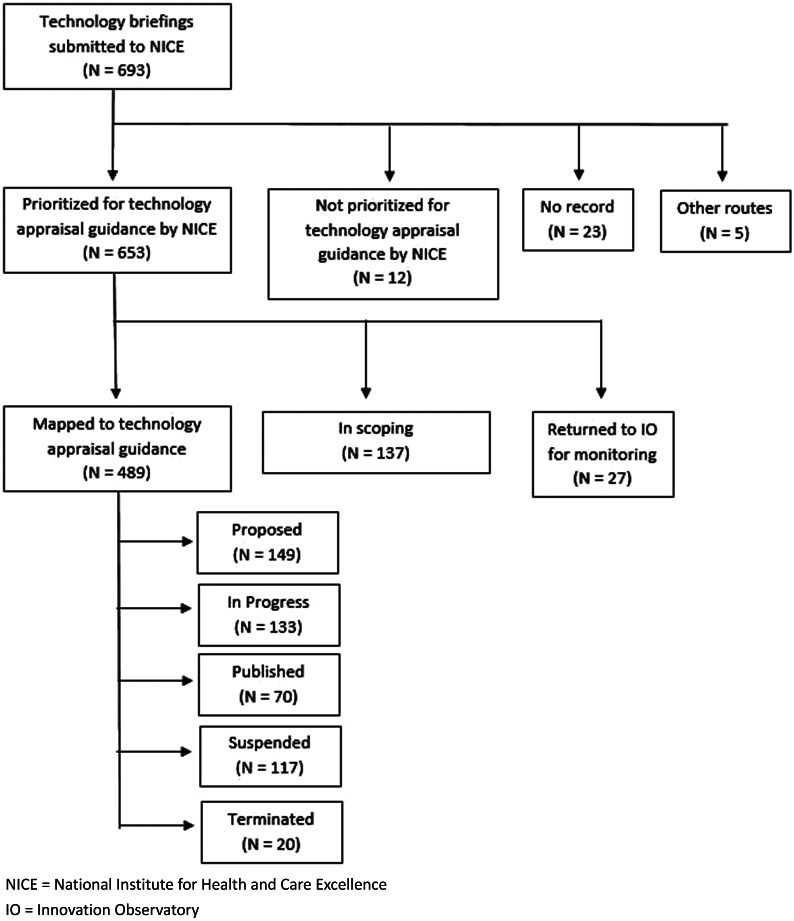
Progression of topics submitted to NICE between April 2017 and October 2021.

Between April 2017 and October 2021, the Innovation Observatory submitted 693 topics for innovative medicines to the NICE TS team. Figure 2 below presents which stage these topics were at in the NICE TA process as of November 2021.

#### Topics prioritized for TA guidance

At the time of this analysis, 489 (70 percent) submitted topics had engaged with the NICE scoping process. Of those, 137 (~20 percent) prioritized topics were still at the NICE TS scoping stage. Twenty-seven (4 percent) topics initially prioritized by NICE were referred back to the Innovation Observatory to be re-opened and further monitored. The most common reason for this was the topic having been submitted to NICE too early. Development of a TA was “proposed” for 149 (21 percent), 133 (19 percent) were “in progress,” seventy (10 percent) were published and 137 (~20 percent) were suspended or terminated.

#### Topics prioritized for TA but suspended or terminated

**Figure 3.**
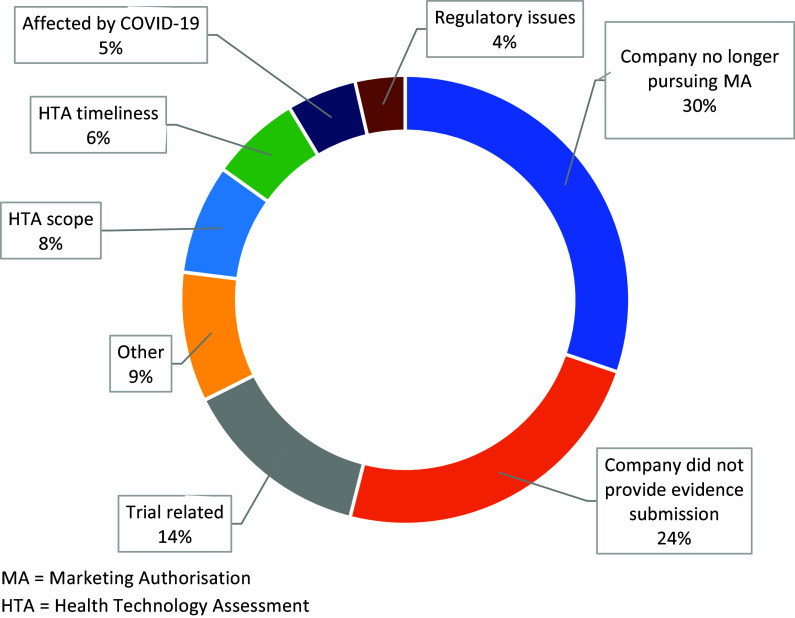
Breakdown of the reasons given for topics being suspended or terminated.

Company decisions, either to withdraw a marketing authorization application (MAA) or not to provide an evidence submission to NICE, were the primary reasons for a topic to be suspended or terminated (Figure 3). Trial-related issues, most commonly clinical trials not meeting primary clinical endpoints were the third largest reason for topics not to proceed in the TA process. Other reasons for suspension or termination included postponement due to coronavirus disease-2019 (COVID-19), topics not aligning with NICE’s HTA timelines, and topics falling out of the HTA scope. For 9 percent of the suspended or terminated topics, the reasons were unknown.

### Analysis of topics submitted

#### Overview of topics submitted by therapeutic area

**Figure 4.**
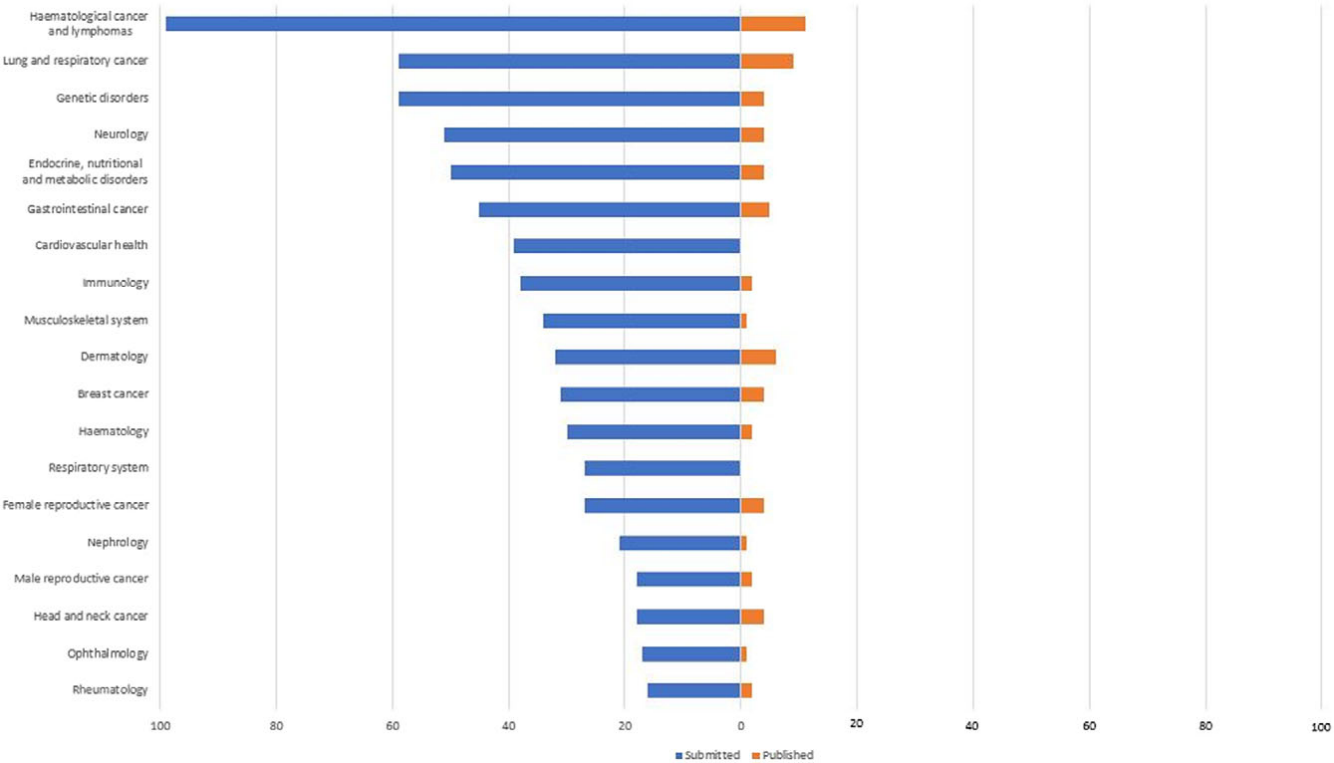
Topics submitted versus topics published by therapeutic area.

The three most common therapeutic areas of the topics submitted to NICE were: hematological cancer and lymphomas (99); lung and respiratory cancer (59); and genetic disorders (59) (Figure 4). There was an equal number of submitted cancer topics (50 percent) and non-cancer topics (50 percent). Likewise, there was a similar proportion of rare disease topics, according to OrphaNet definitions of rare disease, submitted (52 percent) compared to non-rare diseases (48 percent) (10).

#### Overview of topics resulting in a published NICE TA guidance

**Figure 5.**
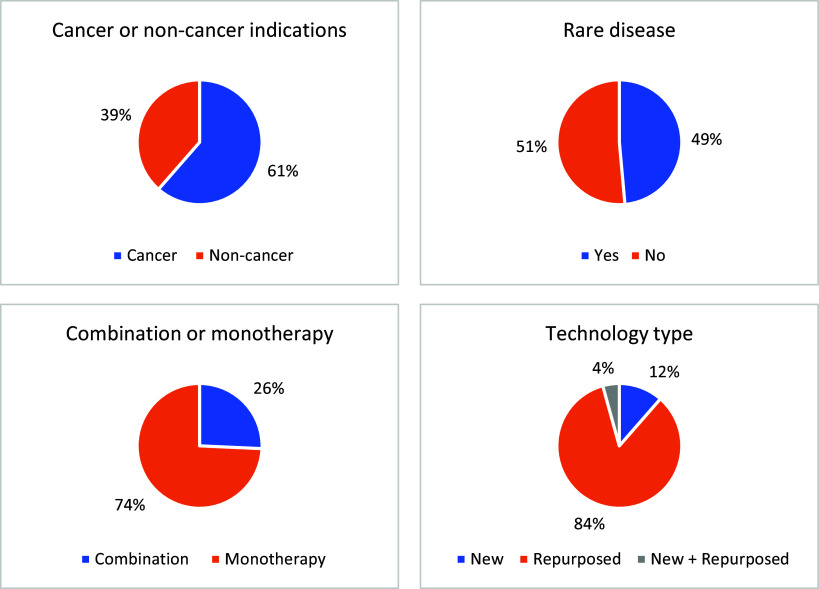
Characteristics of topics that resulted in a published NICE TA guidance.

More extensive analysis was undertaken into the 70 topics which resulted in a published TA (Figure 5). Despite an equal proportion of cancer compared to non-cancer topics being submitted, a greater proportion (61 percent) of published TAs were cancer topics. The most common being hematological cancers and lymphomas (11); lung and respiratory cancers (9); and gastrointestinal cancer (5). Among non-cancer indications, 34 (49 percent) published TAs were for rare disease indications and 36 (51 percent) were for non-rare diseases, which was similar to the overall ratio of topics submitted. Fifty-two (74 percent) topics resulting in a published TA guidance consisted of monotherapy treatments. The vast majority (85 percent), of the topics were repurposed medicines, whereas a minority (15 percent) were for new medicines that had not previously been licensed for any indication at the time of TB submission. A similar trend was observed among 18 published TA topics that consisted of multiple (combination) medicines: 15 (83 percent) of published combination topics consisted entirely of repurposed medicines; three topics (17 percent) involved one repurposed technology in combination with one new technology; no published topics involved a combination where all medicines were new technologies.

## Discussion

The purpose of this project was to examine the HS topics that the Innovation Observatory has notified to NICE for consideration for HTA, to refine HS methods, and to offer some valuable insights into the innovation areas and potential gaps. This exercise has helped gain an understanding of the life cycle of topics following submission to the NICE TS team and identify reasons that can change the trajectory of a topic through its course in the HTA process.

In this study, we found that the majority of topics (94 percent) submitted by the Innovation Observatory for the NICE TA/HST program progressed to prioritization for TA guidance by NICE. The current status of these topics in the NICE HTA program is varied and this study has helped to provide some insight into this variation. While the majority of these topics are still active (proposed, in-scoping, in progress), a significant number, 137 (20 percent), were reported as suspended/terminated mostly because the company had chosen not to provide NICE with an evidence submission or the company was no longer pursuing an MAA. The Innovation Observatory attempts to confirm information regarding potential EMA/MHRA MAA submission dates prior to notifying the topic to NICE; although a lack of clarity and transparency can make this a challenge for some topics. Decisions to not submit/an MAA or evidence to NICE for HTA were taken by companies after the Innovation Observatory had notified the topic to NICE TS team. These post-notification decisions are not routinely captured by the Innovation Observatory’s horizon scanning system. These high-impact unpredicted events or *wild cards* present a challenge to current horizon scanning systems. Further research into the circumstances that surround a company decision to withdraw an application, or disengage with the HTA process, may facilitate earlier identification of signals that indicate which topics may be tagged as uncertain to proceed.

In a few instances, the Innovation Observatory was unable to engage with the company to seek intelligence regarding their regulatory plans for the UK/EU, despite efforts to do so. In such cases, topics were sent to NICE based on information about trial phase, trial location, trial completion dates and news regarding estimated regulatory plans for the UK/EU to facilitate timely HTA; representing proxies for likelihood of an imminent licensing application Our research demonstrates the crucial need for pharmaceutical companies to engage with horizon scanning agencies, such as the Innovation Observatory, as early as possible, with as much intelligence as possible to allow for a smooth HTA process and timely patient access. We suggest that confidential discussions with horizon scanning agencies, prior to topic submission to market access gatekeepers/HTA agencies, such as NICE, would help to ensure timely and efficient HTA and would also reduce the need for pharmaceutical companies to engage with multiple agencies concurrently.

Another objective of this work was to identify emerging trends and gaps in the innovation landscape. Analysis of topics submitted to NICE showed that more than half of the indications were for cancer topics, the majority of which were for lung cancer, hematological malignancies, gastrointestinal cancer, and breast cancer. Lung cancer and breast cancer are among the most common cancers in the UK and in Europe (11–13). The high number of cancer topics might allude to an interest within pharmaceutical companies to develop and launch medicines for more prevalent cancer indications with unmet needs. Additionally, our research showed that more than half of the topics submitted were for rare diseases. This trend is in line with the commercial incentives that regulatory agencies such as EMA and FDA have in place to stimulate medicines development in this area through the orphan designation awards (14;15). Furthermore, the evolution of targeted therapies in the pharmaceutical industry allows the development of investigational treatments for conditions of low prevalence to be a more acceptable business opportunity to investors (14;16).

Over half of the published TA guidance were for rare diseases. It is estimated that there are around 3.5 million people in the UK with rare diseases (17). Stakeholders in the UK recognize that treatments for rare diseases can be life-changing. While there are challenges for clinical trial conduct, and regulatory bodies in assessing these medicines, efforts are underway to improve patient access (17). Based on our findings one may infer that the high number of published TAs for rare diseases may reflect the high levels of unmet need in these diseases and suggest that pharmaceutical companies are making efforts to address this need. The analyses undertaken in this paper suggest also that HTA stakeholders are making progress in improving patient access to innovative medicines for rare diseases; however, according to a 2022 European Commission report 95 percent of rare diseases lack any approved treatments, representing an enormous unmet medical need (18). It is therefore reasonable to infer that a high percentage of TA guidance will continue to be produced for rare conditions for the foreseeable future.

A large majority of the topics that received a published TA guidance involved a medicine that was already marketed for another indication (19). It is common for pharmaceutical companies to trial medicines for indications beyond the initially approved indication (20). This significantly reduces the drug development timeline and saves cost. Additionally, such medicines have already demonstrated safety and pharmacokinetics in previous trials. The MHRA process for licensing repurposed medicines is also shorter and simpler (21). Repurposing medicines already provided to patients in the NHS therefore ensures reducing barriers to patient access in terms of time to market while addressing unmet need. This might explain the high number of repurposed medicines that received a published TA guidance.

## Lessons learned

One of the key factors that would improve the horizon scanning process is greater company engagement and transparency in sharing information with stakeholders. This can be challenging for companies outside of UK/EU. All companies upon contact with NICE regarding HTA are redirected to the Innovation Observatory and asked to engage with UK PharmaScan. This may lead to delays in the HTA process. It is vital that horizon-scanning organizations, including the Innovation Observatory work with industry to establish the best methods of engaging and sharing intelligence early. Horizon-scanning organizations have a clear role in initiating the conversation regarding development and launch of innovative medicines. We suggest that the pharmaceutical industry has a responsibility to engage with these organizations and support early preparedness of the system to consider their medicines.

As clinical development for health technologies constantly evolves, a better understanding of clinical areas of importance to stakeholders might help inform horizon scanning processes and ensure that technologies in development for those clinical areas are captured in a timely manner. Certain companies specialize in specific clinical conditions therefore early engagement might allow for a better understanding of the landscape of upcoming technologies.

## Conclusion

This paper is an initial inquiry into the life cycle of the submitted topics by the Innovation Observatory through the NICE HTA process. The analysis is based on data from England, and the period from November 2021 to the present has not been covered but we feel it highlights issues that are globally generalizable Our analysis showed that the identification of health technologies alongside the alignment of a timely notification to the HTA agency with MA is a fine balance achieved by active engagement with companies and the gathering and interpretation of signals from a vast range of intelligence sources. The regulatory and innovation landscape is constantly moving, alongside advancements in the use of data science and artificial intelligence tools to gather and sort data, it is clear that horizon scanning methods need to evolve to enable a more advanced, less resource-intensive approach to the horizon scanning and HTA process. The capacity for horizon scanning methods to completely eliminate uncertainties about future clinical trial failures or unexpected events such as de-prioritization of company assets is somewhat limited. However, the findings of this analysis suggest that capturing and characterizing that uncertainty in the horizon scanning process may be plausible and of value to further explore. In addition, further exploration and research into clinical trial design and regulatory requirements may offer an opportunity to further characterize some of the drivers of uncertainty and to support differentiating the potential risks for a successful HTA.
